# Genome-Wide Identification of *Trachinotus ovatus* Antimicrobial Peptides and Their Immune Response against Two Pathogen Challenges

**DOI:** 10.3390/md21100505

**Published:** 2023-09-25

**Authors:** Yu Liang, Jin-Min Pan, Ke-Cheng Zhu, Lin Xian, Hua-Yang Guo, Bao-Suo Liu, Nan Zhang, Jing-Wen Yang, Dian-Chang Zhang

**Affiliations:** 1 Key Laboratory of South China Sea Fishery Resources Exploitation and Utilization, Ministry of Agriculture and Rural Affairs, South China Sea Fisheries Research Institute, Chinese Academy of Fishery Sciences, Guangzhou 510300, China; liang_y020@163.com (Y.L.); jimpann97@gmail.com (J.-M.P.); zkc537@163.com (K.-C.Z.); c-xianlin@genomics.cn (L.X.); guohuayang198768@163.com (H.-Y.G.); liubaosuo343@163.com (B.-S.L.); 398730316@163.com (N.Z.); yangjingwen21@126.com (J.-W.Y.); 2 Guangxi Marine Microbial Resources Industrialization Engineering Technology Research Center, Guangxi Key Laboratory for Polysaccharide Materials and Modifications, School of Marine Sciences and Biotechnology, Guangxi Minzu University, Nanning 530008, China; 3 Sanya Tropical Fisheries Research Institute, Sanya 572018, China; 4 Guangdong Provincial Engineer Technology Research Center of Marine Biological Seed Industry, Guangzhou 510300, China

**Keywords:** *Trachinotus ovatus*, antimicrobial peptides (AMPs), *Streptococcus agalactiae*, *Cryptocaryon irritans*, gene expression

## Abstract

Golden pompano, *Trachinotus ovatus*, as a highly nutritious commercially valuable marine fish, has become one of the preferred species for many fish farmers due to its rapid growth, wide adaptability, and ease of feeding and management. However, with the expansion of aquaculture scale, bacterial and parasitic diseases have also become major threats to the golden pompano industry. This study, based on comparative genomics, shows the possibility of preferential evolution of freshwater fish over marine fish by analyzing the phylogenetic relationships and divergence times of 14 marine fish and freshwater fish. Furthermore, we identified antimicrobial peptide genes from 14 species at the genomic level and found that the number of putative antimicrobial peptides may be related to species evolution. Subsequently, we classified the 341 identified AMPs from golden pompano into 38 categories based on the classification provided by the APD3. Among them, TCP represented the highest proportion, accounting for 23.2% of the total, followed by scolopendin, lectin, chemokine, BPTI, and histone-derived peptides. At the same time, the distribution of AMPs in chromosomes varied with type, and covariance analysis showed the frequency of its repeat events. Enrichment analysis and PPI indicated that AMP was mainly concentrated in pathways associated with disease immunity. In addition, our transcriptomic data measured the expression of putative AMPs of golden pompano in 12 normal tissues, as well as in the liver, spleen, and kidney infected with *Streptococcus agalactiae* and skin infected with *Cryptocaryon irritans*. As the infection with *S. agalactiae* and *C. irritans* progressed, we observed tissue specificity in the number and types of responsive AMPs. Positive selection of AMP genes may participate in the immune response through the *MAPK* signaling pathway. The genome-wide identification of antimicrobial peptides in the golden pompano provided a complete database of potential AMPs that can contribute to further understanding the immune mechanisms in pathogens. AMPs were expected to replace traditional antibiotics and be developed into targeted drugs against specific bacterial and parasitic pathogens for more precise and effective treatment to improve aquaculture production.

## 1. Introduction

Antimicrobial peptides (AMPs) are a diverse group of naturally occurring molecules that play a crucial role in the innate immune systems of plants, animals, and humans, serving as the first line of defense against a wide range of pathogens. Ever since the world’s first antibacterial peptide, cecropin, was isolated from northern silkworms by Swedish scientist Hans Bowman [1], one after another, researchers have obtained a variety of antibacterial and bactericidal substances from various organisms in nature. So far, the public database of antimicrobial peptides (APD3, http://aps.unmc.edu/ap/main.php, accessed on 25 September 2022) contains 3167 AMPs from six kingdoms [2]. In addition to extracting natural antimicrobial peptides from biological sources, current methods for obtaining these compounds include chemical synthesis, production through genetic engineering, and enzymatic methods. By designing personalized amino acid sequences based on the known properties of antimicrobial peptides and performing varying degrees of chemical modifications during synthesis as needed, antimicrobial potency can be enhanced [3]. With the rapid development of antimicrobial peptides research, this method of obtaining peptides has become an important means of developing new antibiotics and anti-tumor drugs [4,5].

Antimicrobial peptides are generally short, composed of 7 to 100 amino acid residues [6], with a molecular weight typically less than 10 kDa. They typically carry a positive charge, while some antimicrobial peptides have a negative charge or are uncharged, and exhibit characteristics such as strong alkalinity, thermal stability, and broad-spectrum antimicrobial properties [7]. The secondary structure of antimicrobial peptides, as a key factor in attacking pathogens, often falls into four categories: α-helical, β-sheet, mixed (α-helical/β-sheet), and cyclic structures [3]. The cationic and amphipathic nature of AMPs influences the electrostatic and hydrophobic interactions responsible for peptide binding and insertion into microbial membranes. These interactions compromise membrane integrity, resulting in the leakage of cellular contents and ultimately leading to cell death, thereby achieving antimicrobial effects [8,9]. Beyond the antimicrobial mechanism involving cell membrane permeabilization, AMPs are also capable of penetrating bacterial cells and interacting with specific intracellular targets, thereby inhibiting the synthesis of nucleic acids and proteins [10]. AMPs not only exhibit antimicrobial properties but also possess antiviral, antifungal, antiparasitic, and immunomodulatory activities [5]. These functions render antimicrobial peptides highly promising for widespread applications in the aquaculture industry.

The aquaculture sector has expanded significantly and holds a crucial position in global food security. Nevertheless, production in aquaculture faces numerous challenges due to various diseases and parasites [11]. *Streptococcus agalactiae* and *Cryptocaryon irritans* are two common pathogens in the aquaculture industry, causing severe health issues in fish. *S. agalactiae* is a Gram-positive bacterium that primarily infects the blood circulatory system of fish, leading to conditions such as septicemia, myositis, and abscesses. *C. irritans*, an ectoparasite, infests the skin and gill tissues of fish, causing symptoms such as itching, rubbing, and respiratory distress [11,12,13]. Due to the severe damage caused by pathogen and parasite infections to the production and economic benefits of aquaculture, the overuse of antibiotics has led to the development of drug resistance in fish [14]. Consequently, researchers have begun focusing on fish-derived antimicrobial peptides as novel therapeutic alternatives to antibiotics.

According to the literature, fish antimicrobial peptides are mainly divided into five major families: hepcidins, β-defensins, cathelicidins, histone-derived peptides, and fish-specific piscidins [15]. Fish antimicrobial peptides have similar functions as other biological antimicrobial peptides. Besides antibacterial or bacteriostatic activities, they also have antifungal, antiviral, and antiparasitic effects. In addition, fish antimicrobial peptides also have immunomodulatory effects and can be used as vaccine adjuvants [16]. Fish antibacterial peptides were first discovered in the 1980s, and then many kinds of fish antibacterial peptides were reported one after another [17]. Research shows that the synthetic *Amatitlania nigrofasciata* hepcidin peptide significantly improved the survival rate of *S. agalactiae* and *Vibrio vulnificus*-infected zebrafish [18]. β-defensin-1 plays a role in the infection of rainbow trout with viral hemorrhagic septicemia virus (VHSV) [19]. The protective effects of cathelicidins (Cath and codCath2) on zebrafish infected by bacteria were studied. It was found that cathelicidins could kill bacteria by inducing bacterial membrane permeabilization and cell destruction and showed effective broad-spectrum antibacterial activity [10]. Bergsson obtained from the skin mucus of Atlantic cod, an antibacterial peptide similar to histone H2B, had strong inhibitory effects on *Bacillus megaterium*, *Candida albicans*, and *Escherichia coli* [20]. Tilapia piscidin 3 overexpression in zebrafish is resistant to *S. agalactiae* infection and effectively inhibits the pro-inflammatory response [21]. *C. irritans* infection impacts the marine fish industry, especially China’s *Larimichthys crocea*, which combats the infection by producing piscidin-5-like molecules [22]. The histone-1-derived peptide in *Larimichthys crocea* exhibits a time-dependent expression pattern after *C. irritans* infection, demonstrating broad-spectrum antimicrobial activity against various microbial strains [23].

*Trachinotus ovatus*, commonly known as the golden pompano, is a fish species belonging to the Carangidae family and the Trachinotus genus. It is widely distributed along the coasts of the Indian and Pacific Oceans, particularly in the South China Sea and the East China Sea. As a highly valuable marine fish with strong adaptability, it can thrive and reproduce in various aquatic environments [24,25]. However, intensive farming methods and high-density environments make golden pompano susceptible to various bacterial diseases, while long-term use of antibiotics can lead to pathogen resistance and drug residue accumulation, raising food safety concerns [26]. Fish rely more heavily on innate immunity, so fish antimicrobial peptides can serve as potential defensive weapons against emerging devastating diseases. Based on genome-wide identification of fish antimicrobial peptide genes, this research direction has yielded results in species such as lined seahorse, giant grouper, amphibious mudskipper, catfish, and black rockfish [5,9,27,28,29]. However, only a few antimicrobial peptide genes have been reported in golden pompano, such as NK-lysin, LEAP-2, CCL4, and β-defensin [26,30,31,32]. Research on the whole-genome antimicrobial peptide genes of golden pompano has not yet been discovered. Therefore, conducting research on the identification, enrichment pathways, chromosomal localization, PPI (protein–protein interaction), covariance analysis, and antimicrobial spectrum of AMPs in golden pompano based on whole-genome and then exploring the mechanism of antibacterial immunity will lay the theoretical foundation for the future development of antimicrobial peptide drugs targeting the golden pompano, thereby improving the disease resistance and economic benefits of the aquaculture industry and promoting the healthy and sustainable development of the fish farming industry.

## 2. Results

### 2.1. Phylogenetic Analysis and Divergence Time Estimation

**Figure 1:**
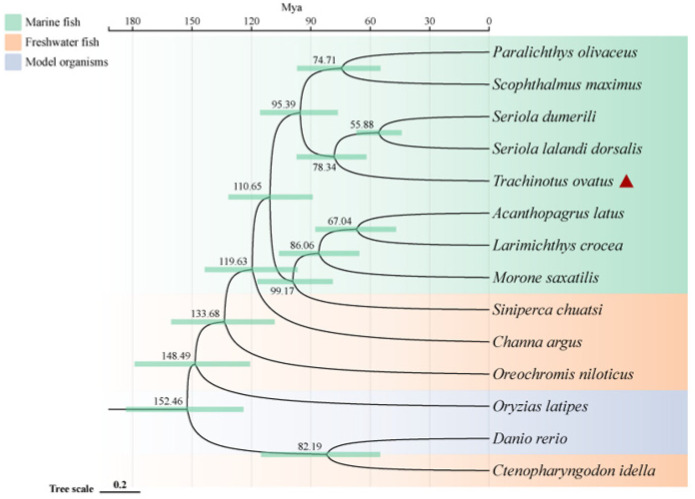
Phylogenetic relationships of *Trachinotus ovatus* and other species. The phylogenetic tree is plotted by the online software ITOL V6 (https://itol.embl.de, accessed on 25 September 2022). The red triangle symbol is the highlight of the *T. ovatus*, which is the subject of this study.

To investigate the phylogenetic relationship of the golden pompano within teleosts and its genetic differences with other marine and freshwater fish, we conducted a comparative genomics analysis. The results show that a total of 2669 single-copy homologous genes were obtained by homology clustering analysis of the genomes of 14 species (*Trachinotus ovatus, Acanthopagrus latus*, *Larimichthys crocea*, *Seriola lalandi dorsalis*, *Scophthalmus maximus*, *Seriola dumerili*, *Paralichthys olivaceus*, *Morone saxatilis*, *Channa argus*, *Siniperca chuatsi*, *Ctenopharyngodon Idella*, *Oryzias latipes*, *Oreochromis niloticus*, and *Danio rerio*). At the same time, we also discovered 1284 genes that are unique to the golden pompano. Enrichment analysis indicates that these genes primarily participate in various pathways including pathways of neurodegeneration-multiple diseases, pathways in cancer, pathogenic Escherichia coli infection, thyroid hormone signaling pathway, and MAPK signaling pathway. Additionally, the phylogenetic tree reveals that marine and freshwater fish (including two model species of freshwater fish) are divided into two distinct clades. Interestingly, the golden pompano, along with *S. lalandi* dorsalis and *S. dumerili*, forms a sister group that clusters with *P. olivaceus* and *S. maximus*. Divergence time analysis indicates that marine fish underwent speciation later than most freshwater fish, all occurring after 119 million years ago (Mya). The earliest divergence time for the golden pompano, *S. lalandi* dorsalis, and *S. dumerili* is around 78 Mya (Figure 1).

### 2.2. Identification of Potential AMPs and Comparison of Teleosts

**Figure 2:**
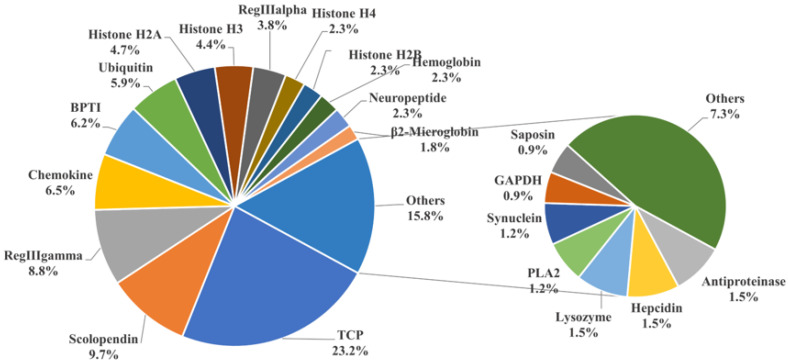
Classification of AMP genes in *Trachinotus ovatus*. Different colors represent different classes of AMP genes, and the numbers represent the proportion of the total number of AMP genes in *T. ovatus*. The result is displayed with one decimal place.

To identify potential AMPs, we used the entire genome of golden pompano as a database for comparison. We cross-referenced this with the 3167 AMP sequences collected from the APD antimicrobial peptide database using TBLASTN. Ultimately, this resulted in the identification of 341 potential AMP genes in the golden pompano (Supplementary Material File S2). Each putative AMP gene was assigned a new identifier, and, using the classification scheme from the APD database, they were grouped into 38 categories. The thrombin (TCP) gene group represented the highest proportion, accounting for 23.2% of the total, followed by scolopendin, lectin (RegIIIalpha, RegIIIgamma), chemokine, BPTI, and histone-derived peptide groups. Each of these also made up more than 5% of the total gene copy numbers. The counts of AMP genes such as hemoglobin, neuropeptide, β2-microglobin, hepcidin, and lysozyme were relatively fewer, each accounting for around 2% of the total (Figure 2). Employing the same homology-based approach, we carried out the identification and classification of putative antimicrobial peptides in both marine and freshwater fishes, including species such as *A. latus*, *L. crocea*, *S. lalandi dorsalis*, *S. maximus*, *S. dumerili*, *P. olivaceus*, *M. saxatilis*, *C. argus*, *S. chuatsi*, *O. niloticus*, *C. idella*, *O. latipes*, and *D. rerio* (Table 1). The results showed that the number of TCP did not vary significantly among marine fish. However, in freshwater species such as *C. idella*, *O. niloticus*, and *D. rerio*, the number exceeded 100 genes. There was not a significant difference in the number of scolopendin, BPTI, ubiquitin, and neuropeptide genes among teleosts. In addition, it was observed that the maximum counts for lectin (RegIIIgamma, RegIIIalpha) and histone-derived peptides were found in *O. niloticus*, *C. idella*, and *D. rerio*, respectively. Regarding the total count of AMP genes, the highest quantity was observed in *O. niloticus* (722), while the lowest was seen in *P. olivaceus* (306). The number of antimicrobial peptide genes in golden pompano was comparable to that in other marine fish species, all falling within the range of 300 to 500.

### 2.3. Positive Selection of AMP Genes

To delve deeper into how the genes in *T. ovatus* adapt to its specific ecological environment and biological needs, we placed special emphasis on positive selection analysis. The results indicated that out of the 2669 single-copy homologous genes identified through OrthoFinder homology alignment clustering, 838 single-copy genes in *T. ovatus* exhibited positive selection compared to other fish species, as determined by PAML analysis (using default parameters) with a significance level of *p* < 0.05. Moreover, among the 341 identified AMP genes, five genes that underwent positive selection were classified as antimicrobial peptides. These genes are *hpn*, *tmprss6*, *macroh2a2*, *ubtd1*, and *flnc*, with detailed information provided in Table 2. This analysis underscores the evolutionary significance of these genes in *T. ovatus* and their potential adaptive advantages in countering microbial threats.

### 2.4. Localization and Enrichment Analysis of AMP Genes

**Figure 3:**
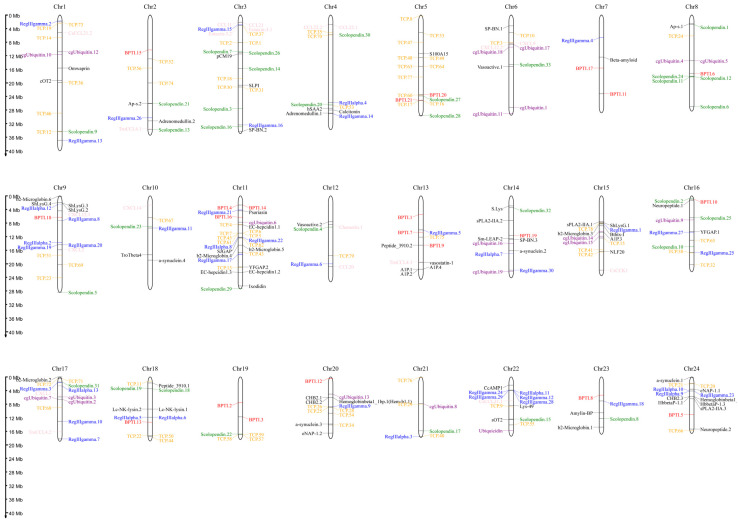
Distribution of 341 antimicrobial peptide genes (AMPs) on 24 chromosomes in *Trachinotus ovatus*. The picture was drawn by the online tool MG2C v2.1 (mg2c.iask.in/mg2c_v2.1/). TCP is marked in orange, lectin in blue, BPTI in red, scolopendin in green, histone-derived in gray, ubiquitin in purple, chemokine in pink, and the rest of the AMP genes in black.

**Figure 4:**
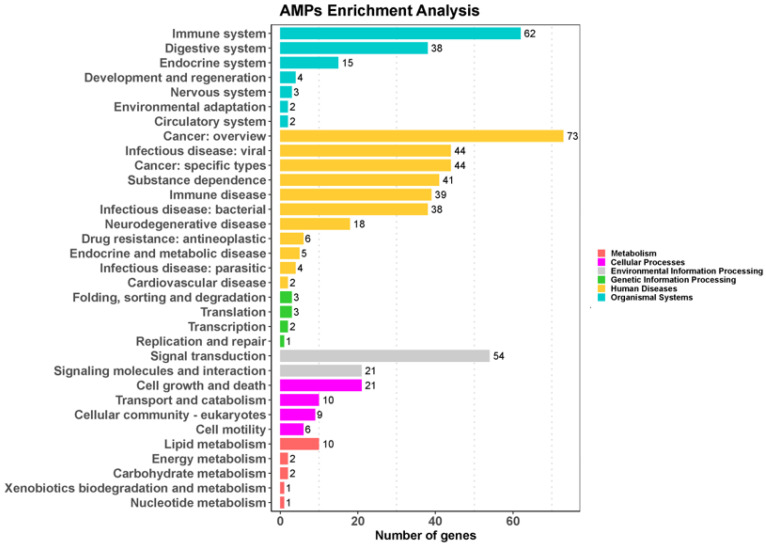
KEGG metabolic pathway annotation of 341 AMPs in *Trachinotus ovatus*. Data were analyzed and visualized by ClusterProfiler 4.0 and ggplot2 in R (*p*-value of 0.05 for KEGG analysis). Different colors represent different primary pathways, and the number following each secondary pathway is the number of antimicrobial peptide genes enriched for that pathway.

In order to characterize the AMP genes in golden pompano, 341 AMPs were mapped onto 24 chromosomes (Figure 3). The distribution of the AMP genes varied across each chromosome, both in terms of numbers and types. The fewest hits were found on Chr7, with only five identified AMP genes. In contrast, Chr9 and Chr11 held the highest quantity of AMP genes, with counts reaching up to 30 and 28, respectively. The TCP, which was the most numerous, was identified on all chromosomes except for Chr7, Chr14, and Chr23, where it was absent. However, it was primarily localized on Chr5 and Chr11. Distribution patterns of histone-derived genes differed across distinct subunits yet exhibited a predominant concentration within Chr9 and Chr21. Subsequently, an enrichment analysis of 341 AMP genes was performed using the Kyoto Encyclopedia of Genes and Genomes (KEGG), which revealed that 158 of these AMP genes were enriched in 34 KEGG items (Figure 4). Among them, the two major pathway categories with a larger proportion of AMP genes were human diseases and organismal systems. The pathways that showed the highest number of gene enrichment were “cancer: overview” (73 genes) and immune system (62 genes), respectively. Other representative pathways included infectious disease: viral (44 genes), cancer: specific types (44 genes), substance dependence (41 genes), infectious disease: bacterial (38 genes), digestive system (38 genes), and signal transduction (54 genes), etc. Moreover, we noticed that most of the genes enriched in the “cancer: overview” and immune system pathways were histone-derived peptides, implying their indispensable role in the organism’s antimicrobial functions

### 2.5. PPI Analysis and Covariance Analysis of AMPs

**Figure 5:**
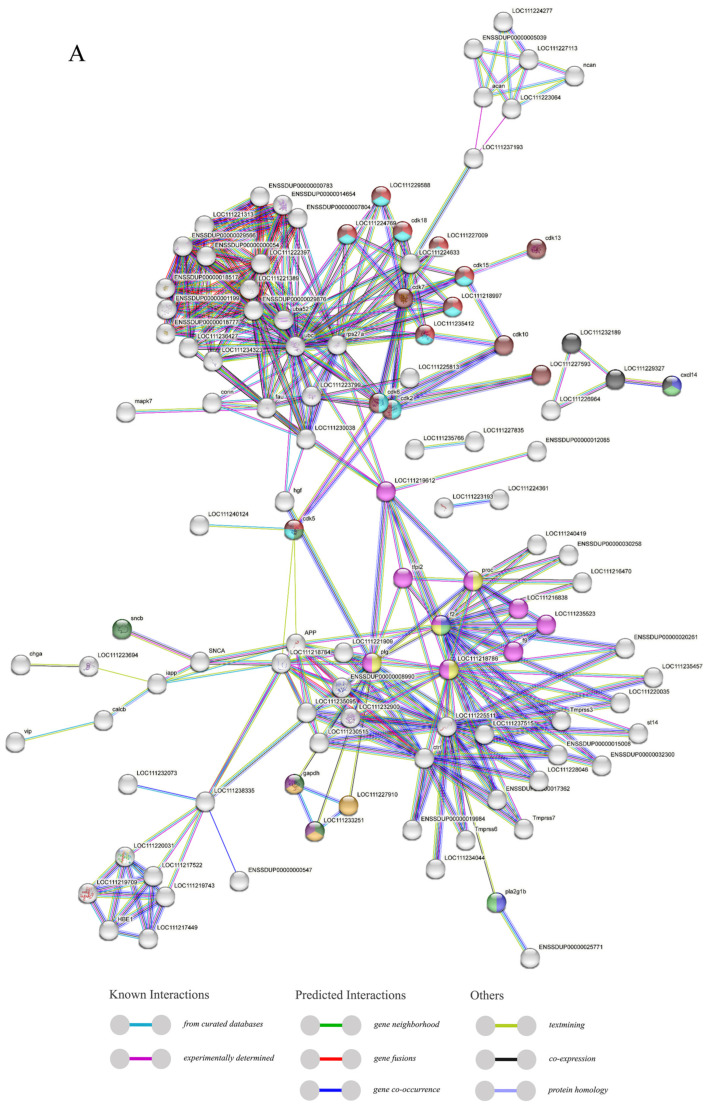

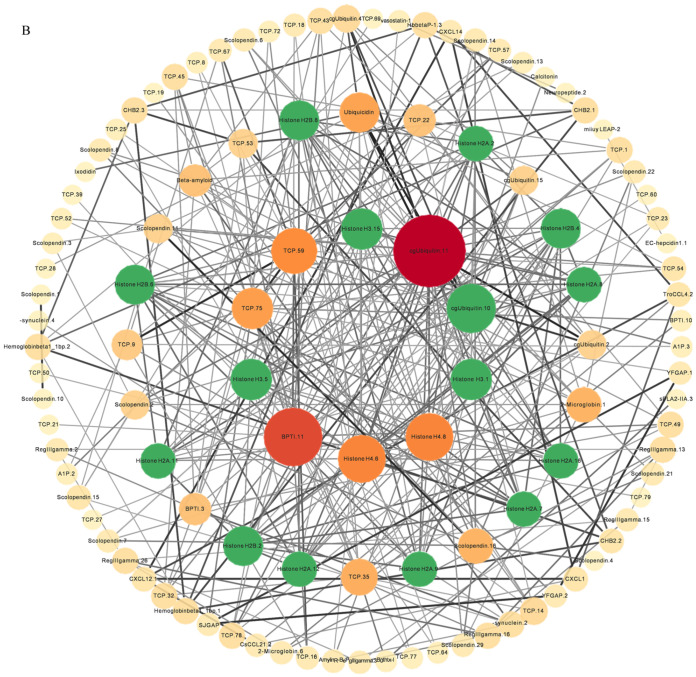
The protein–protein interactions between 341 members of antimicrobial peptides in *Trachinotus ovatus*. (**A**) A network of 341 AMP proteins with their parent and subordinate proteins. This figure was drawn via STRING 12.0 (https://STRING-db.org/, accessed on 25 September 2022). It has employed a confidence level of 0.40, derived from text mining, experimental data, databases, co-expression patterns, and neighborhood sources in lines of different colors. The same color within the circle indicates that these genes are enriched in the same KEGG pathway. (**B**) The interaction network of 341 AMP proteins distributed by DC value. This graph was analyzed by Cytoscape and visualized by Circos (default parameter selection). The colors range from dark red to light yellow, indicating DC values from high to low, and the green areas are selected HUB gene interaction maps. The abbreviation labels in the figure are supplemented in File S3.

**Figure 6:**
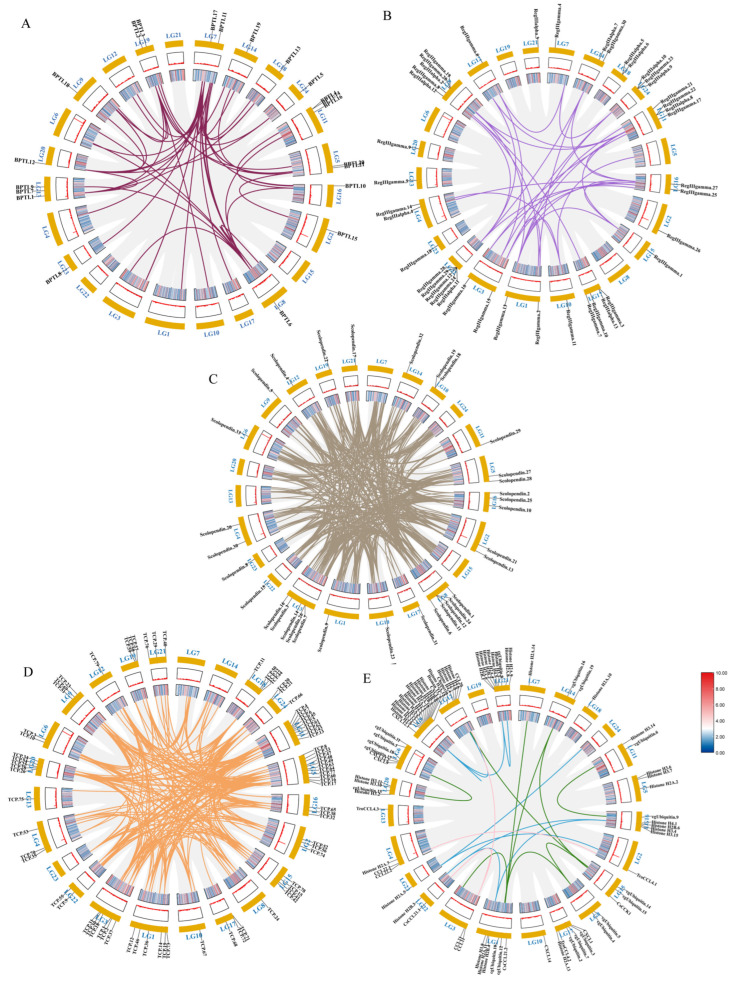
Intraspecific covariance of 341 antimicrobial peptide genes in *Trachinotus ovatus*. Grey lines indicate all covariate blocks in the *T. ovatus* genome, and different colored lines indicate pairs of genes from different species gene families. Co-linear blocks of BPTI (**A**), lectin (**B**), scolopendin (**C**), TCP (**D**), chemokine, histone-derived, and ubiquitin pairs (**E**). Chromosome numbers are labelled at the top of each chromosome. Heat maps and line plots indicate the gene density of each chromosome in *T. ovatus*.

Through the analysis of the PPI network, the protein–protein interactions of the AMP genes were shown (Figure 5) (File S3). The results indicate that the antimicrobial peptide protein was closely associated with biological processes such as the regulation of transcription involved in the G1/S transition of the mitotic cell cycle (cdk18, cdk5, cdk15), the antimicrobial humoral immune response mediated by antimicrobial peptides (cxcl14, f2, pla2g1b), the negative regulation of blood coagulation (plg, proc, f2), and blood coagulation (tfpi2, f2, f9, proc, plg) (Figure 5A). Meanwhile, in the network diagram, the core node with the highest degree centrality was the ubiquitin protein, which has the most network connections, followed by BPTI and histone-related proteins. This suggests that they might play roles in different parts of the network. Proteins enclosed in the green circle are HUB genes identified through MCODE analysis. They constitute a complex module composed of tightly connected protein nodes and might have synergistic functions. The main members are histone- and ubiquitin-related proteins (Figure 5B). Additionally, the collinearity analysis of the antimicrobial peptide gene suggests its involvement in various types of replication events and regulatory functions (Figure 6). BPTI homologous gene pairs were identified on every chromosome (Figure 6A). For lectin, apart from chromosomes 19, 6, 18, 21, 8, 2, 20, 13, and 23 where no significant gene pairs were observed, gene pairs were present on all other chromosomes (Figure 6B). Preliminary observations indicate that the frequency of gene duplication events for both is moderate. Scolopendin and TCP exhibit a higher number of gene pairs on each chromosome, some of which might form homologous gene pairs with other unnamed genes (Figure 6C,D). Both might have undergone numerous gene duplication or fragment repetition events. The result reveals that repeated sequences of genes related to histone, ubiquitin, and chemokine are more prevalent on chromosomes, yet fewer gene pairs are formed (Figure 6E).

### 2.6. Expression Profile of AMP Genes in Healthy Tissues

**Figure 7:**
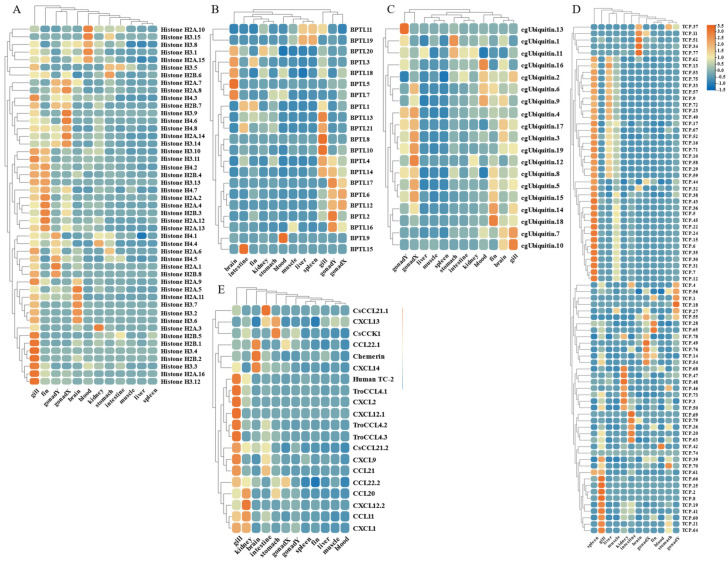
Expression patterns of AMP genes in *Trachinotus ovatus* among different tissues. (**A**) Histone-derived; (**B**) BPTI; (**C**) ubiquitin; (**D**) TCP; (**E**) chemokine. Different colors indicate different expression values that were scaled to standard deviations. Red color indicates upregulation and blue indicates downregulation when compared to RPKM.

**Figure 8:**
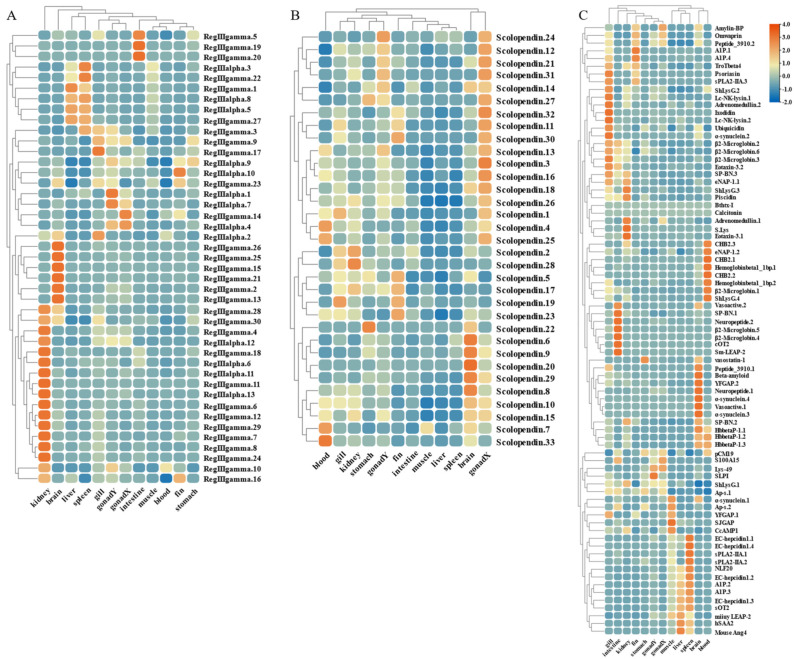
Expression patterns of AMP genes in *Trachinotus ovatus* among different tissues. (**A**) Lectin; (**B**) scolopendin; (**C**) other genes. Different colors indicate different expression values that were scaled to standard deviations. Red color indicates upregulation and blue indicates downregulation when compared to RPKM.

By analyzing the existing transcriptomic dataset of our group, the tissue transcriptomic expression profiles of these AMP genes in golden pompano were mapped. In general, AMP genes are widely expressed in all healthy tissues (intestine, liver, muscle, brain, spleen, fin, gill, kidney, stomach, blood, gonad-Y, and gonad-X), and the same genes are expressed at different levels due to tissue variations (Figure 7 and Figure 8). Specifically, a higher overall expression level of histone genes was observed in gill, fin, and gonad. Additionally, elevated expression of histone H2A and histone H3 was observed in the brain and blood. The brain, gill, and gonad tissues exhibited high expression levels of both BPTI and ubiquitin. The expression levels of TCP were relatively high in spleen, gill, and liver and increased in kidney, intestine, and gonad. Chemokine was also expressed at different subunits but mainly at higher levels in gill and kidney. In the kidney and brain, lectins (RegIIIgamma, RegIIIalpha) display higher expression levels, while their expression is also observed in the liver, spleen, and gill. Conversely, scolopendin demonstrates predominant expression in the gonad-X and brain. For other types of AMP, we found that multiple copies may exhibit different expression patterns. For example, β2-Microglobin.2, β2-Microglobin.6, and β2-Microglobin.3 are more highly expressed in gill, whereas β2-Microglobin.4 and β2-Microglobin.5 are slightly more highly expressed in intestine. In addition, distinct expression patterns were observed among different subtypes of the same AMP gene. Hemoglobinbeta1_1bp displayed higher expression levels in blood, and HbbetaP-1 exhibited higher expression levels in the brain and blood, whereas CHB2 showed predominant expression in blood. It is worth noting that all three subtypes belong to the hemoglobin family.

### 2.7. Transcriptome Quantification of AMP Genes by S. agalactiae and C. irritans Infection

**Figure 9:**
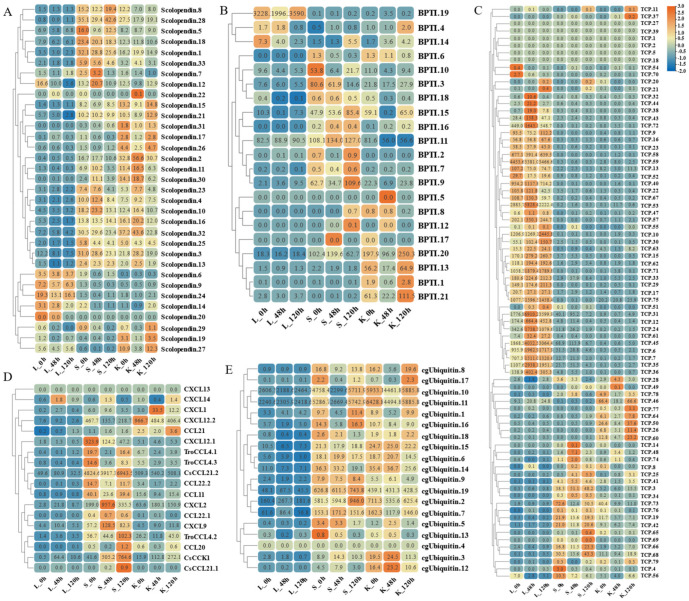
Expression patterns of AMP genes in *Trachinotus ovatus* after *S. agalactiae* infection. (**A**) Scolopendin; (**B**) BPTI; (**C**) TCP; (**D**) chemokine; (**E**) ubiquitin. Different colors indicate different expression values scaled to standard deviations. Red color indicates upregulation and blue indicates downregulation when compared to the normal conditions. Values of relative fold change are marked on the heat maps.

**Figure 10:**
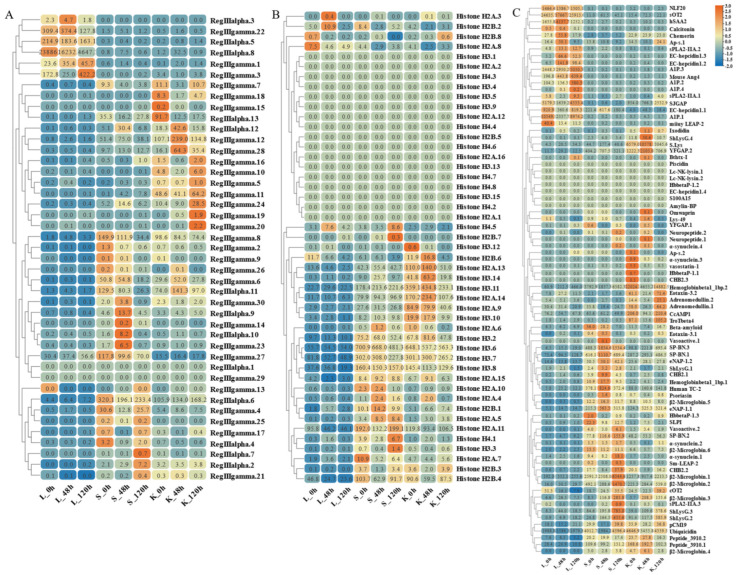
Expression patterns of AMP genes in *Trachinotus ovatus* after *S. agalactiae* infection. (**A**) Lectin; (**B**) histone-derived; (**C**) other genes. Different colors indicate different expression values scaled to standard deviations. Red color indicates upregulation and blue indicates downregulation when compared to the normal conditions. Values of relative fold change are marked on the heat maps.

The widespread expression of these AMP genes is concentrated in gill, gonad, and immune tissues such as kidney, intestine, and spleen. Therefore, based on transcriptome sequencing data, we investigated the possible immune response of the AMP genes in liver, spleen, and kidney after infection by *S. agalactiae* (Figure 9 and Figure 10). Following bacterial attack, scolopendin and ubiquitin showed massive high expression in both spleen and kidney compared to liver. Compared to the control group, Scolpentin.7, Scolpentin.12, Scolpentin.4, and Scolpentin.10 in the spleen were upregulated 3.2-, 20.7-, 12.4-, and 23.2-fold, respectively, after 48 h of infection. In the kidney, significant upregulation of Scopentin.2, Scopentin.11, Scopentin.30, and Scopentin.32 occurred during the early stage of infection (48 h), while significant upregulation of Scopentin.15, Scopentin.21, and Scopentin.26 occurred during the late stage of infection (96 h). Furthermore, Ubiquitin.19, Ubiquitin.2, Ubiquitin.3, and Ubiquitin.12 were highly induced in the spleen at 96 h post-infection, while in the kidney they showed significant induction at 48 h post-infection (Figure 9A,E). High expression levels of BPIT.15, BPIT.11, and BPIT.9, as well as BPIT.20, BPIT.13, and BPIT.21, were observed in the spleen and kidney 96 h after infection (Figure 9B). Next, in the TCP types, the liver exhibited more pronounced changes in expression compared to the spleen and kidney. The majority of TCP gene copies displayed elevated expression levels in the liver as the infection progressed. Additionally, TCP.66 and TCP.68, as well as TCP.50, TCP.26, and TCP.60, demonstrated significant upregulation in the spleen and kidney at 96 h post-infection, with fold changes of 23.3-, 43.3-, 37.6-, 11.4-, and 23.2-fold, respectively (Figure 9C). CXCL2 and CXCL9 in chemokine were 957.6-fold and 128.5-fold upregulated in the spleen at 48 h of infection; by 96 h, the higher expression levels were CsCCL21.2, TroCCL4.2, and CsCCK1 (Figure 9D). Moreover, at 48 h post-infection, the spleen exhibited significant upregulation in the expression of RegIIIγ.30, RegIIIγ.23, RegIIIalpha.9, and RegIIIalpha.10. In the kidney, the expression levels of RegIIIγ.12 and RegIIIγ.28 were notably upregulated by 239-fold and 64.8-fold, at 48 h post-infection; however, not until 96 h later, substantial upregulation was primarily observed for RegIIIγ.11 and RegIIIγ.29, with fold changes of 64.2-fold and 28.5-fold (Figure 10A). For the histone type, more copy genes were also upregulated in spleen and kidney than in liver. Some copy genes of histone H2B, histone H2A, and histone H3 were significantly induced in kidney at 48 h of infection (Figure 10B). Among other types of AMP genes, various types of AMP manifested differential tissue-specific expression patterns with increasing duration of infection (Figure 10C). After 48 h of infection, HSAA2, Ap-s.1, sPLA2-IIA.2, EC-hepcidin1.2, and EC-hepcidin1.3 were mainly upregulated in liver expression. On the other hand, at 96 h, A1P.2, A1P.3, sPLA2-IIA.1, SJGAP, and EC-hepcidin1.1 demonstrated prominent upregulation in liver expression. Furthermore, an increase in expression levels was observed in spleen, primarily after 48 h of infection, for SP-BN.1, SP-BN.3, eNAP-1.2, CHB2.1, human TC-2, Psoriasin, and eNAP-1.1. Conversely, significant induction in kidney, after 96 h of infection, was observed for SP-BN.2, α-synuclein.1, CHB2.2, β ShLysG.4, S.Lys, and YFGAP.2, among others, following 48 h of infection.

**Figure 11:**
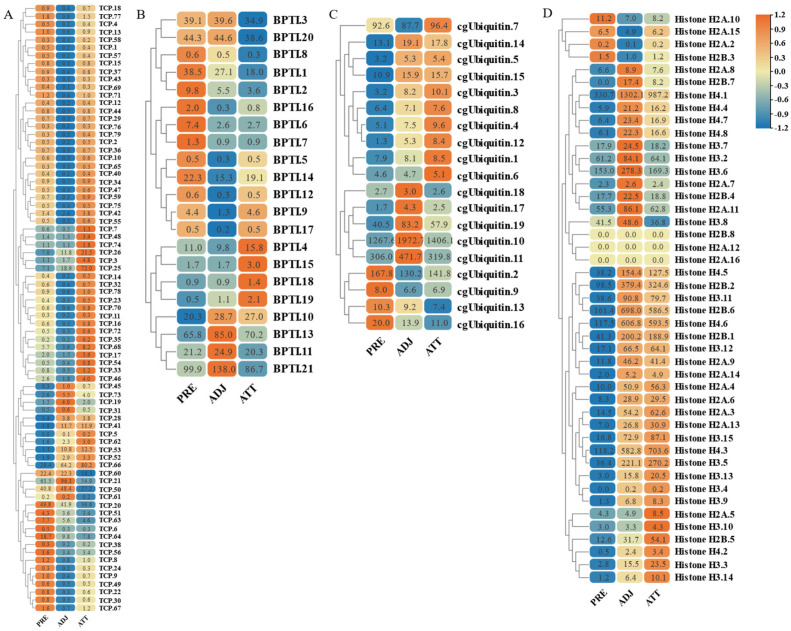
Expression patterns of AMP genes in *Trachinotus ovatus* after *C. irritans* infection. (**A**) TCP; (**B**) BPTI; (**C**) ubiquitin; (**D**) histone-derived. Different colors indicate different expression values scaled to standard deviations. Red color indicates upregulation and blue indicates downregulation when compared to the normal conditions. Values of relative fold change are marked on the heat maps.

**Figure 12:**
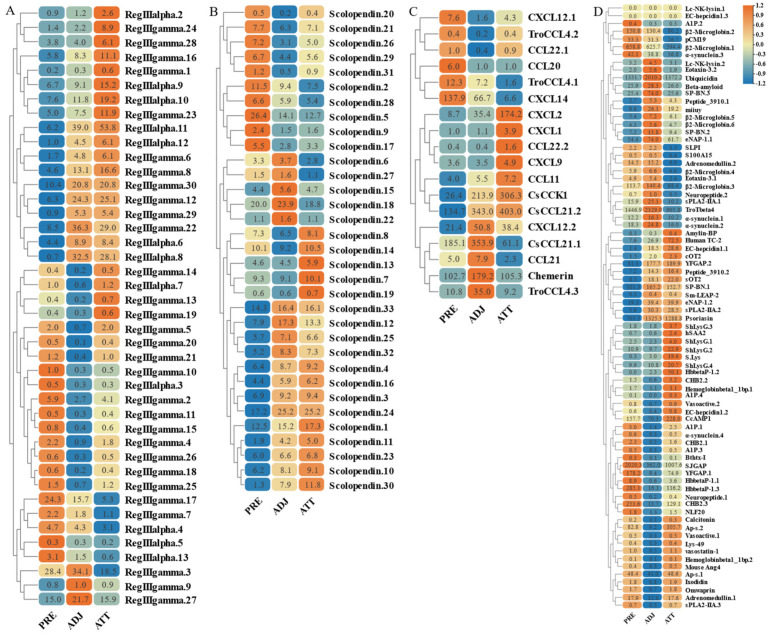
Expression patterns of AMP genes in *Trachinotus ovatus* after *C. irritans* infection. (**A**) Lectin; (**B**) scolopendin; (**C**) chemokine; (**D**) other genes. Different colors indicate different expression values scaled to standard deviations. Red color indicates upregulation and blue indicates downregulation when compared to the normal conditions. Values of relative fold change are marked on the heat maps.

On the other hand, based on transcriptome sequencing data, we also investigated the expression pattern of AMP genes in the skin of *C. irritans*-infected golden pompano (Figure 11 and Figure 12). Significant upregulation of gene expression was observed at the ATT among the TCP types. There were substantial changes in TCP.25, TCP.26, TCP.53, and TCP.66, which were upregulated 72-, 21.5-, 12.5-, and 80.2-fold, respectively, compared to the control group (Figure 11A). BPTI expression levels were mainly increased at BPTI.10, BPTI.13, BPTI.11, and BPTI.21 in the ADJ, but BPTI.4, BPTI.15, BPTI.18, and BPTI.21 were relatively upregulated in the ATT (Figure 11B). Ubiquitin types were also observed to be heavily induced mainly at the ATT, where cgUbiquitin.7, cgUbiquitin.14, cgUbiquitin15, cgUbiquitin.3, and cgUbiquitin.4 were upregulated by 96.4-, 17.8-, and 15.7-fold. Next, cgUbiquitin.18, cgUbiquitin.17, cgUbiquitin.19, cgUbiquitin.10, and cgUbiquitin.11 were also found to be induced at the ADJ (Figure 11C). Histone-related peptides were found to be widely expressed at both ADJ and ATT sites and were differentially elevated between copies of different subfamilies (Figure 11D). In addition, the genes belonging to the lectin family exhibited upregulation at the ATT. Particularly, RegIIIgamma.8, RegIIIgamma.12, RegIIIgamma.30, and RegIIIgamma.22 showed significant upregulation with fold increases of 16.6, 25.1, 20.8, and 29, respectively. Similarly, RegIIIalpha.9, RegIIIalpha.10, RegIIIalpha.11, and RegIIIalpha.8 were also upregulated, with fold increases of 15.2, 19.2, 53.8, and 28.1, respectively (Figure 12A). Various copies of scolopendin were differentially induced at ADJ and ATT sites. Scolopendin.33, Scolopendin.12, and Scolopendin.18 were found to be induced at sites adjacent to the infection, whereas Scolopendin.8, Scolopendin.14, and Scolopendin.13 were only found to be significantly upregulated at the site of ATT (Figure 12B). Chemokines originating from different subfamilies were differentially expressed at the ADJ and ATT sites (Figure 12C). Compared to other AMP genes, specific genes such as SP-BN.3, eNAP-1.1, Eotaxin-3.2, β2-Microglobin.5, Lc-NK-lysin.2, and α-synuclein.3 were found to be induced and upregulated at the ADJ site. On the other hand, genes such as ShLysG, HbbetaP-1.2, CHB2.2, CcAMP1, and Ap-s.2 exhibited upregulation in expression levels at the ATT site. Furthermore, some AMP genes demonstrated high expression levels in both sites, including YFGAP.2, sOT2, SP-BN.1, eNAP-1.2, sPLA2-IIA.2, and Psoriasin (Figure 12D).

## 3. Discussion

Scientific research confirms that the evolution of species is strongly driven by genomic variation, which reveals species identity and drives evolutionary patterns. Delving into genomic data will not only help us to unravel species identity but also advance our understanding of the laws of biological evolution [5,28]. With the continual advancement of sequencing technologies, the demand for high-quality genome assembly and interpretation in the aquaculture industry is growing increasingly. The large amount of published genomic data in teleosts provides an important reference for our understanding of the evolutionary relationships between species [9]. However, AMPs are essential components of the biodefence system, both for active resistance to pathogens and for interacting with the adaptive immune system. In particular, within the teleost fishes, AMPs have demonstrated broad-spectrum antimicrobial activity against various pathogens, including those affecting both humans and fish [7,29]. With the identification and characterization of an increasing number of fish AMPs, approximately 60 specific AMPs have been discovered in fish species [15]. Therefore, this study focused on the golden pompano, one of the economically important aquaculture fish species in the South China Sea. Using a genomic approach, we screened putative antimicrobial peptides (AMPs) and explored the genetic evolutionary level, classification characteristics, chromosomal localization, enrichment pathway analysis, and immune response triggered by pathogen infections in golden pompano.

### 3.1. Comparative Genomic Analysis and Evolutionary Insights

To characterize the antimicrobial peptide genes in the genome, we conducted comparative genomics analysis of *T. ovatus* with 13 marine and freshwater fish species including *A. latus*, *L. crocea*, *S. lalandi dorsalis*, *S. maximus*, *S. dumerili*, *P. olivaceus*, *M. saxatilis*, *C. argus*, *S. chuatsi*, *O. niloticus*, *C. Idella*, *O. latipes*, and *D. rerio* and found that freshwater fishes mostly differentiated preferentially over marine fishes [33]. In contrast, freshwater fish had already emerged prior to 152 Mya, while golden pomfret did not differentiate until 78 Mya. This could be due to the existence of a greater number of barriers in freshwater habitats, as compared to marine ones, which could potentially lead to more frequent instances of allopatric speciation events. It has been reported that freshwater lineages often exhibit higher rates of speciation and extinction than their marine counterparts [34]. Faster evolution to adapt to environmental stresses may have contributed to the larger genomes of freshwater fish such as *O. niloticus* and *D. rerio* than marine fish such as *T. ovatus* and *S. dumerili*. Subsequent genome-wide identification of AMP genes from these 14 species showed that they have conserved evolutionary features [9]. *T. ovatus* was identified with 341 putative AMPs, not very different from the number of putative antimicrobial peptide genes in *P. olivaceus* and *S. maximus*, which diverged at almost the same time, both being in the region of three hundred. In contrast, *A. latus* and *L. crocea* have a larger number of putative antimicrobial peptides than *T. ovatus*, probably because they face greater environmental stress and need to increase the copy number of genes associated with resistance to environmental stress in order to evolve faster [35,36]. In addition, we found that *C. Idella*, *O. niloticus*, and *D. rerio* in freshwater fish had significantly higher numbers of antimicrobial peptides, upwards of 600 to 700, compared to other fish, mainly due to increased copy numbers of immune-related genes such as thrombin, lectin, and β2-microglobin. The reason could be due to species differentiation, which enabled them to colonize more efficiently in the complex freshwater environment [37].

### 3.2. Classification and Significance of AMPs in Teleosts

By classifying the AMP genes of 14 teleost fish species, we found that thrombin, scolopendin, histone-derived peptides, and lectin were at the top of the list, followed by chemokine, BPTI, and ubiquitin. Similar results were found for *Oreochromis aureus*, *Hippocampus erectus*, and *Amphibious Mudskippers* [27,29,38]. Currently, thrombin, as the main type of AMP, has revealed that host defense peptides from the C-terminal part of human thrombin have a broad inhibitory effect on multiple sepsis pathologies [39]. Researchers have found that scolopendin 1 demonstrates antimicrobial activity without causing hemolysis in human erythrocytes and additionally induces apoptosis in *Candida albicans* [40,41]. In addition, we identified 22 AMP genes associated with chemokine in *T. ovatus*. The antimicrobial activity of TroCCL4 against *Escherichia coli* and *Edwardsiella tarda* has been validated. Furthermore, it has been demonstrated to possess the capability to stimulate leukocytes and macrophages [30]. The results of the KEGG enrichment analysis showed that the majority of antimicrobial peptide genes enriched in the “cancer: overview” pathway were in the histone family. Research indicates that the complete histone H2A of zebrafish possessed antimicrobial properties against the Gram-negative bacterium *Edwardsiella piscicida* in vivo, though its efficacy was somewhat constrained [42]. Nonetheless, it has been discovered that histone variants and histone-derivative peptides, because of their structural changes and sequence modifications, exhibit enhanced antibacterial properties [43,44,45]. Lectin families, which include RegIIIalpha and RegIIIgamma, feature prominently in the enriched pathways. These families derive from regenerating islet-derived proteins and constitute a significant portion of secreted C-type lectins [9]. Reports have confirmed that they can kill Gram-positive bacteria by forming hexameric membrane-permeabilizing oligomeric pores [46]. The genetic diversity of species contributes to the variance in the copy number of antimicrobial peptide genes within their bodies. This study identifies the antimicrobial peptides in the golden pompano and, in doing so, lays a theoretical foundation for the future development of antimicrobial peptide drugs for treating diseases in this species [7,9,11].

### 3.3. PPI Analysis and Covariance Analysis of AMPs

To further explore the regulatory mechanisms of the antimicrobial peptide gene, interactions between the antimicrobial peptide genes were connected based on PPI (protein–protein interaction) network analysis, which was crucial for predicting the activity of most proteins [47]. *Cxcl14*, *f2*, and *pla2g1b* were among the key factors involved in the antimicrobial humoral immune response mediated by antimicrobial peptides. As a member of the chemokine family, *cxcl14* frequently binds to its specific receptor, activating signal transduction and triggering an antimicrobial immune response [47]. *F2* was a thrombin-related gene, with the host defense peptides from its C-terminal part found to exhibit broad inhibitory effects on multiple sepsis pathologies [39]. On the other hand, *pla2g1b* was a member of phospholipase A2, and its capability against both Gram-negative and Gram-positive bacteria is notably evident [48,49]. Next, ubiquitin was a protein that contained the ubiquitin domain and played a pivotal role in the immune system [50]. As the core node in degree centrality, it interacted with most of the other antimicrobial peptide gene proteins, suggesting its generic role in antimicrobial immunity. Among these, the histone protein, identified as a key HUB factor, was found to form a strongly associated interactive network with other members of its family. This might have indicated that, under certain conditions, they participated in biological processes through mutual interactions and, in specific splice forms, were activated. Through various cascading signal transductions, they activated downstream factors, achieving antimicrobial efficacy [45,51,52].

Additionally, collinearity analysis results showed that the golden pompano antimicrobial peptide gene had high collinearity within the species, but its repeated sequences displayed varying degrees of replication events due to differences. The varying degrees of fragment repetition might have been related to the gene’s historical evolutionary process and selective pressure [53]. Notably, this study found that the antimicrobial peptide gene formed homologous gene pairs with other unnamed genes. This possibly suggests its synergistic participation in biological functions, such as antimicrobial immunity, signal transduction, and resistance to abiotic stress, with genes that have similar biological functions [54,55]. However, the results revealed that the copy number formed by replication events did not correlate directly with the formation of gene pairs, which might have been related to the complexity of the gene family. The specific reasons need further analysis and validation.

### 3.4. Immune Responses to Pathogen Infections

In recent years, infections caused by *S. agalactiae* have been occurring frequently in teleost fishes, and golden pompano is no exception. Immune mucosal tissues such as the liver, spleen, and head kidney serve as the first line of defense for the host against pathogens [56,57]. Investigating the immune expression mechanisms of AMPs in these tissues of golden pompano will provide a solid foundation for the development of novel antimicrobial agents for aquaculture. The research results revealed that these AMP genes showed varying levels of high expression in the mucosal immune system of golden pompano infected with *S. agalactiae*. For example, EC-hepcindin1 was found to accumulate in the liver over the course of infection, leading to an increase in its expression level. Hepcidin, as an immune regulatory factor synthesized in the liver, plays a role in both iron homeostasis regulation and antimicrobial activity [9]. Researchers found that *Amatitlania nigrofasciata* attacked by lipopolysaccharides resulted in a significant increase in hepcidin transcript expression in the liver and a significant antibacterial effect against *S. agalactiae* [18]. Another study indicated that a derived peptide, TroHepc2-22, was synthesized from the mature peptide segment of *T. ovatus* hepcidin-2. This peptide demonstrated excellent antimicrobial capabilities against both Gram-negative (*Vibrio harveyi* and *Edwardsiella piscicida*) and Gram-positive (*Staphylococcus aureus* and *S. agalactiae*) bacteria. It is presumed that the antimicrobial activity of TroHepc2-22 was exerted by disrupting the bacterial membrane structure and subsequently hydrolyzing bacterial gDNA [58]. Another widely studied AMP, LEAP-2, a member of the hepcidin family, has also been found to be highly expressed in the spleen and kidney following infection with *S. agalactiae*. Some researchers have found that upon induction by *Aeromonas hydrophila*, the expression of the grass carp LEAP-2 gene was significantly upregulated in the liver, gill, skin, muscle, spleen, blood, head kidney, heart, and intestine [59]. Mature peptides or recombinant proteins synthesized from fish sources have been verified to possess antimicrobial activity against both Gram-positive and Gram-negative bacteria [26,60]. In addition, the expression of defensin (eNAP-1.1, eNAP-1.2) was revealed to be significantly upregulated in the post-infected spleen, and similar expression changes were also identified in *Acipenser dabryanus* [27] and *Liza haematocheila* [61]. β-Defensin, a small group of cationic antimicrobial peptides, has a structure of six conserved cysteine residues that are important in exerting their antimicrobial and antiviral effects. Researchers have found that *TroBD* expression was significantly upregulated in the head kidney and spleen following *Vibrio harveyi* or viral nervous necrosis virus (VNNV) infection of golden pompano. Moreover, overexpression of *TroBD* significantly inhibits bacterial infection, even at high salt concentrations, and may exhibit antimicrobial activity if the concentration of β-defensins is sufficiently high [32]. In fish infected with Singapore grouper iridovirus (SGIV) or VNNV, the synthetic peptide *Ec-defensin* interacts directly with viral particles or persists locally on the surfaces of the viral envelope or target cell surfaces, exerting an impact on the target cells. This interaction helps to block early viral replication and achieve antimicrobial efficacy [62]. A further study based on transcriptomic data found that CXCL was significantly upregulated in the spleen and kidney of tilapia infected by *S. agalactiae* after 6 h [63]. Their results indicate that upon Streptococcus infection in fish, macrophages and B lymphocytes within the body migrate to the inflammatory zone in immune tissues mediated by CXCL10 and C-C chemokine ligand 19 and subsequently undergo proliferation and differentiation through downstream factors activation. This process enables them to exert their antimicrobial immune functions [56,57]. Our results demonstrate that the upregulation of CXCL and CCL chemokine types in the spleen may also involve similar antimicrobial immune mechanisms. Overall, the antimicrobial peptides were differentially expressed in different tissues following *S. agalactiae* infection of *T. ovatus* and different copies of the same AMP genes also showed different expression levels. Differentially expressed AMP genes could be selected as a first choice for further validation of their activity and development into novel antimicrobial drugs.

On the other hand, *C. irritans*, the causative agent of marine white spot disease (Cryptocaryonosis), is a holotrichous ciliate that invades the gills, eyes, and skin of the host. During a stage in its life cycle, it burrows into the gills, skin, and pterygiophore, where it feeds on the host’s epithelial layers. This invasion can result in respiratory impairment, osmotic stress, and secondary bacterial infections, ultimately leading to the death of the host [13]. According to reports, L-amino acid oxidases (LAAOs) and matrix metalloproteinase 9 (MMP9) were found to be upregulated in gene expression in the skin of *T. ovatus* infected with *C. irritans*. This observation suggests their inherent resistance to parasites and their potential role in the host’s defense mechanism against the pathogen [64,65]. However, this study found that most AMPs were also stimulated by *C. irritans* to stimulate antiparasitic capacity. β2-Microglobulin (β2M) is a small protein that binds to major histocompatibility complex class I (MHC I) molecules, forming a trimer complex known as *pMHC-I*, which functions as an immunomodulator [66]. A study of *Epinephelus coioides* found that genes involved in the MHC II antigen presentation pathway were upregulated in skin and parotid mucosa after infection with *C. irritans*, but the regulatory mechanism has not been determined [67]. MHC I contained the same α-chain as MHC II, and it may act on cells infected with parasites through cellular immune mechanisms. Therefore, upon stimulation by pathogen infection, its expression is highly induced, leading to an increase in β2M expression [68]. Another study found that hemoglobin-beta homologous antimicrobial peptide (HbβP-1) derived from *Ictalurus punctatus* was induced and upregulated in response to *Ichthyophthirius multifiliis* stimulation, which was consistent with our findings [69]. The mucoid capsule secreted in response to *HbβP-1* appeared visibly similar to the cyst formed by normal trophonts during their differentiation to form a tomont. When trophonts leave the host, they immediately form a mucoid cyst prior to undergoing division as a tomont. This reduces the degree of parasitic infection of the organism by creating specific lethal damage to the trophonts. Its hemoglobin-derived mutant peptide and hemoglobin β-chain-related peptide have also been shown to have anti-Gram-positive and Gram-negative effects in *Tegillarca granosa* and *Katsuwonus pelamis*, respectively [70,71]. The research also revealed that the Lc-NK-lysin.2 was upregulated in the ADJ region under the influence of *C. irritans*. In one study [72], it was found that treatment of individuals infected with *Philasterides dicentrarchi* using a synthetic peptide, Nkl71-100, synthesized based on the NK-lysin gene sequence from *Scophthalmus maximus*, resulted in a significant reduction in parasite load compared to the control group. It has been demonstrated that Nk-lysin functions by inserting its α-helices into the target cell membrane, thereby causing membrane disruption and exerting antimicrobial efficacy against pathogens. In conclusion, based on our data, a number of AMPs were found to be affected by *C. irritans* infection in golden pompano, causing an immune response. By further investigating these inducible AMP genes, we can gain a deeper understanding of their mechanisms of action, regulatory pathways, and interactions with other immune molecules. This will pave the way for new directions in the development of therapeutic strategies for antiviral and antibacterial treatments.

### 3.5. Analysis of Selective Pressure on AMP Genes

The analysis results of selective pressure indicate that the genes of *hpn*, *tmprss6*, and *ubtd1* are all related to disease immunity. Hepsin (hpn), also known as transmembrane protease serine 1 (TMPRSS1), and TMPRSS6 both encode type II transmembrane serine proteases [73]. Although they belong to the same family, their biological functions are different. Hepsin is associated with some diseases, especially cancer, while TMPRSS6 is mainly involved in iron metabolism and iron deficiency anemia [74,75]. Similarly, they occur mainly in the liver. Ubiquitin domain-containing protein 1 (ubtd1) is a highly evolutionarily conserved protein, primarily encoded by the *ubtd1* gene, which contains a ubiquitin structural domain. In disease genesis, *ubtd1* has also been found to be abnormally expressed in a variety of cancers, including hepatocellular carcinoma, gastric cancer, and lung cancer, suggesting that it may play a role in tumorigenesis and progression [50]. This study found that under the infection of *S. agalactiae* and *C. irritans*, the immune response occurrence of *hpn* in the liver was stronger than that in the spleen, head kidney, and skin, but it showed a negative correlation with response time. Although the expression of *ubtd1* presented slight fluctuations with the intensification of pathogen stimulation, no strong upregulation of expression was observed. This suggests that when pathogen stimulation occurs, the *hpn* gene in the liver may respond first, regulating interactions with other genes to affect cell proliferation, EMT/metastasis, inflammation, and tyrosine kinase signaling pathways through the hydrolysis of proteases [76]. Low *ubtd1* expression induces *RhoA* activation, increases cell traction forces, and activates YAP signaling via *ROCK2* fostering cancer cell aggressiveness [50,77]. The low expression of *hpn* and *ubtd1* also indirectly suggested that *S. agalactiae* and *C. irritans* stimulus infections did not cause carcinogenic effects on golden pompano at the experimental level of this study. The *tmprss6* gene has been found to participate in inflammatory responses through negative regulation with *hepcidin*. The inhibition of *tmprss6* by decreasing *STAT5* phosphorylation allowed inflammatory stimuli to increase ferredoxin secretion to regulate iron homeostasis and immunity [75]. Upon bacterial stimulation, *hepcidin* activated the mechanism of nutritional immunity by regulating the concentration of iron ions in the blood, thereby exerting an antibacterial immune function [78]. The upregulation of *tmprss6* expression in the later stages of infection may be related to the nutritional consumption of the body involved in immunity and the absorption and utilization of iron. In addition, the results of selection pressure analysis also found that *macroh2a2* and *flnc* were affected by positive environmental selection. The core histone macro-H2A.2 (macroh2a2) is one of the histone H2A variants; its polypeptide has been confirmed to have direct antibacterial activity and primarily participates in the killing action against Pseudomonas aeruginosa through its alpha-helical structure [79]. Under the infection of *S. agalactiae* and *C. irritans*, *macroh2a* was found to be largely activated in the kidney and skin. It may induce the transcription of many antibacterial genes and *MHC*-related genes, enhance the sensitivity to inflammatory cytokines, and indirectly participate in the antibacterial immune response through *NLR* and *NF-κB* signaling pathways [42]. As a cytoskeletal protein, filamin-C (flnc) mainly carries out signal transduction in muscle tissues and participates in the maintenance of key cellular functions. Therefore, it is rarely found to be significantly upregulated in immune tissues under pathogen stimulation [80]. However, when subjected to excessive stress, it may be activated to assist the migration of immune cells and indirectly trigger an immune response by participating in the *MAPK* signaling pathway [81]. In summary, positively selected genes in golden pompano are mainly involved in disease, antimicrobial, and signaling aspects and are involved in all aspects of the immune response induced by pathogen challenge.

## 4. Materials and Methods

### 4.1. Data Collection

A total of 3167 AMP sequences demonstrating antimicrobial activity were downloaded from the public Antimicrobial Peptides Database (APD3, http://aps.unmc.edu/AP/main.php, accessed on 25 September 2022) (File S1). Genome data of *Acanthopagrus latus* (GCF_904848185.1), *Larimichthys crocea* (GCF_000972845.2), *Seriola lalandi dorsalis* (GCF_002814215.2), *Scophthalmus maximus* (GCF_013347765.1), *Seriola dumerili* (GCF_002260705.1), *Paralichthys olivaceus* (GCF_001970005.1), *Morone saxatilis* (GCF_004916995.1), *Channa argus* (GCA_004786185.1), *Siniperca chuatsi* (GCF_020085105.1), *Ctenopharyngodon Idella* (GCF_019924925.1), *Oryzias latipes* (GCF_002234675.1), *Oreochromis niloticus* (GCF_001858045.2), and *Danio rerio* (GCF_000002035.6) were downloaded from National Center for Biotechnology Information (NCBI) database (https://www.ncbi.nlm.nih.gov/, accessed on 25 September 2022). Our research group has provided the original transcriptome dataset covering 12 healthy tissues (intestine, liver, muscle, brain, spleen, fin, gill, kidney, stomach, blood, gonad-Y, and gonad-X) of the golden pompano, as reported in our previous publication, which includes the assembled genome scaffold and annotated gene set of *Trachinotus ovatus* [25].

### 4.2. Phylogenetic Analysis and Divergence Time Estimation

To elucidate the phylogenetic location of golden pompano, we performed phylogenetic analysis of single-copy homologous genes obtained from the genome of golden pompano with the genomes of marine fish such as *A. latus*, *L. crocea*, *S. lalandi dorsalis*, *S. maximus*, *S. dumerili*, *P. olivaceus*, and *M. saxatilis* and freshwater fish such as *C. argus*, *S. chuatsi*, *O. niloticus*, and *C. idella*, as well as model organisms including *O. latipes* and *D. rerio*, by homologous clustering with the OrthoFinder 2.0 program [82]. We enriched the unique gene of golden pompano, and the single-copy homologous genes of each species are matched one by one with a MAFFT, which then forms a super-alignment matrix with default parameters. Then, a phylogenetic tree of default parameters is constructed with RAXML (V8.2.12). The three calibration times—*T. ovatus* and *S. dumerili* (52.4–57.9 Mya), *S. chuatsi* and *A. latus* (83.2–142.9 Mya), and *O. niloticus* and *C. idella* (180.0–251.1 Mya)—were used as well as phylogenetic trees to estimate the divergence times of 14 species. For this calculation, we used Mcmctree program from the PAML v4.9 package.

### 4.3. Identification and Analysis of Potential AMPs

Standard homology searches were used to predict AMPs. For each gene set’s data, an individual index was created using the makeblastdb command. Then, potential AMPs were identified by aligning the active AMP sequences collected with the gene set sequences, based on TBLASTN sequence similarity (e-value: 1e-5). We utilized an internal script to filter out alignment hits with a ratio of less than 0.5 and to eliminate any redundant results. Additionally, this script was employed to extract both the aligned region sequences and the full-length gene sequences from the archived subject sequences. The predicted AMPs obtained were annotated according to the classification in the ADP3 database, with detailed information acquired for each hit. Subsequently, we employed the same method to predict AMPs in the gene sets of marine fish such as *A. latus*, *L. crocea*, *S. lalandi dorsalis*, *S. maximus*, *S. dumerili*, *P. olivaceus*, and *M. saxatilis* and freshwater fish such as *C. argus*, *S. chuatsi*, and *C. Idella*, as well as model organisms including *O. latipes* and *O. niloticus*. Then, these results were compared with golden pompano to compare the difference in antimicrobial peptides between different marine fish and freshwater fish.

### 4.4. Screening of Positive Selection Genes

Based on the single-copy homologous genes of 14 species, the selection pressure of antimicrobial peptide genes was analyzed. The ratio of nonsynonymous substitution (Ka) to synonymous substitution (Ks), namely the ω value (Ka/Ks), is usually used to represent the selection pressure in the analysis of sequences. When ω = 1, it means neutral evolution, that is, it is not under selection pressure; when 0 < ω < 1, it means negative selection (also known as purifying selection), that is, the intensity of the constraint. Only when ω > 1 does it mean positive selection, under positive selection pressure. In this research, the software PAML V.4.9 [83] and its codeml tool were utilized to estimate the Ka/Ks (omega) values. The branch-site model was chosen due to its better alignment with the actual scenarios of intricate species divergence processes. For each gene, codeml was used to calculate the likelihood value [84], and this value was further utilized to compute the likelihood ratio statistic. Subsequently, a chi-square test was applied to obtain the corresponding *p*-value. To control for false discovery rate (FDR) across the entire genome, all *p*-values were adjusted accordingly. Genes with FDR values less than 0.05 were selected as the final candidate positively selected genes [85].

### 4.5. Localization and Enrichment Analysis of AMP Genes

We investigated the clustering patterns of AMP genes in specific chromosomal regions by conducting chromosomal position analysis of the AMP genes. To determine the chromosomal location of each gene and the length of the 24 chromosomes, we extracted data from the annotation file for the golden pompano, provided by our research group. We then used the online tool MG2C v2.1 (mg2c.iask.in/mg2c_v2.1/, accessed on 15 June 2023.) [86] to visualize the location of each gene on the chromosome. Additionally, we identified potential AMPs and performed differential analysis of KEGG signal pathways related to AMP genes using the ClusterProfiler 4.0 in R 4.2.2 package. The enrichment analysis results were visualized using the ggplot2 package, with gene count as the reference.

### 4.6. PPI (Protein–Protein Interaction) Analysis and Covariance Analysis of AMPs

The interactions between 341 members of antimicrobial peptides were forecasted via STRING 12.0 (https://STRING-db.org/) [87]. An assessment of protein–protein interaction portrayal was conducted, employing a confidence level of 0.40, derived from text mining, experimental data, databases, co-expression patterns, and neighborhood sources. Then, the results were visualized and screened for core genes using Cytoscape v3.10.0 software (degree cutoff = 2, node score cutoff = 0.2, k-core = 2, and max.depth = 100) [88]. In addition, the Molecular Complex Detection (MCODE) plugin in Cytoscape v3.10.0 was used for the screen modules of the PPI network in Cytoscape v3.10.0 for HUB genes. In order to investigate the duplication events of these AMP gene sequences within the species, the MCscanX (2012) package [89] was employed to identify collinear blocks within the genome of *T. ovatus*. Subsequently, the covariation among the AMP genes within the *T. ovatus* genome was extracted and modeled. As a result, covariance circle maps of these genes were constructed using the Circos-0.69-9 software [90].

### 4.7. Healthy Tissues Extraction and Pathogens Challenge

To explore the expression levels of the predicted AMP genes in response to *S. agalactiae* and *C. irritans* infections, golden pompano specimens were obtained from the Shenzhen Experimental Base of the South China Sea Fisheries Research Institute of the Chinese Academy of Aquatic Sciences. Individuals with an average body length of 30 cm were cultured in a flow-through system and acclimated at 28 °C for one week prior to the experiment. Tissues were collected from healthy golden pompano, including intestine, liver, muscle, brain, spleen, fin, gill, kidney, stomach, blood, gonad-Y, and gonad-X, with three replicates per group and stored at −80 °C. Animal research was approved by the Committee of the South China Sea Fisheries Research Institute, Chinese Academy of Fisheries Sciences (no. SCSFRI96-253), and performed in accordance with the applicable standards.

The used of *S. agalactiae* was isolated from the diseased golden pompano at the Shenzhen Base of the South China Sea Fisheries Research Institute. After purification and identification, it was stored at −80 °C. Prior to infection, the bacteria were cultured in brain–heart infusion (BHI) liquid medium for 24 h at 28 °C and 140× *g* on a shaker. The BHI liquid culture was centrifuged at 6200× *g* for 8 min, and the precipitate was recovered. A cell suspension with five concentration gradients (1.0 × 10^10^, 1.0 × 10^9^, 1.0 × 10^8^, 1.0 × 10^7^, 1.0 × 10^6^ CFU/mL) was then diluted and plated. The concentration of each gradient was calculated by counting the colony-forming units (CFUs) per milliliter. The final 120 h half-lethal concentration (120 h LD50) of 2.0 × 10^7^ CFU/fish was obtained by multiplying the dilution of the PBS solution. For the infection experiment, six 150 L (140 L of water) aquariums were used. Three hundred healthy pomfret fish were randomly assigned to a control group and an infection group, with three replicates per group. The infection group was injected with 200 μL of bacterial solution at a concentration of 2.0 × 107 CFU/fish, while the control group was injected with 200 μL of sterile PBS. Specimens were collected at 0 h, 48 h, and 96 h post-infection. The liver, spleen, and kidney of nine randomly selected fish in each group were sampled at each time point, and three fish samples were pooled into one sample (fish were anesthetized with 40 mg/L clove oil prior to sampling). The samples were stored at −80 °C.

The experiment involved infecting fish with *C. irritans* at a concentration of 8000/L. After 48 h of infection, skin tissues were collected from nine fish each from the infected group and the control group. The skin tissue in the control group was uninfected (prior to infection, PRE), while the skin tissue in the infected group was divided into two categories: the skin attached to the infected area (attached, ATT) and the skin adjacent to the infected area (adjacent, ADJ). All samples were frozen at −80 °C for subsequent transcriptome sequencing.

### 4.8. Gene Expression Analysis of AMPs

Transcriptome sequencing analysis was performed on tissue samples from three biological replicates each of the control and infected groups from both infection experiments. Methods for the *S. agalactiae* infection experiment were adapted from researchers in our research group [91]. Data were obtained from NCBI’s Bioproject, accession number PRJNA871979. Other transcriptome data were obtained from unpublished papers and unpublished data. Starting with total RNA, small RNA libraries were constructed using the Small RNA Sample Pre Kit, and strand-specific libraries were built using ribosomal RNA depletion (for circRNA library construction, the linear RNA depletion method was used). After filtering the transcriptome sequencing data, HISAT2 was used to build the index of the reference genome and align the paired-end clean reads with the reference genome. The RPKM values and expression patterns of these AMP genes were analyzed. The screening criteria for differentially expressed genes were |log2(FoldChange)| > 1 and padj ≤ 0.05. The Benjamini–Hochberg method was used to adjust the resulting p-value to control for the false discovery rate. Then, the expression data were visualized using a heat map in TBTOOLS [92].

## 5. Conclusions

Based on comparative genomics, this study conducted a phylogenetic analysis of golden pompano and 13 marine and freshwater fish species, in conjunction with divergence time. It was found that most freshwater fish diverged earlier than marine fish. Antimicrobial peptides are crucial molecules in the innate immune system, and in this study we identified AMP genes in 14 teleost fishes using genomics approaches, revealing a decrease in the number of AMP genes with evolution. The 341 putative AMPs identified in golden pompano were found to be scattered in 24 chromosomes, and their repeat sequences were conserved on different chromosomes. They were involved in the biological process of humoral immunity and related to disease. Through analyzing the gene expression levels of these AMP genes in healthy tissues and different tissues after infection with *S. agalactiae* and *C. irritans*, it was discovered that most AMP genes exhibited high expression at varying degrees of stimulation. Positive selection of AMP genes may participate in immune response through the *MAPK* signaling pathway. These findings provide crucial clues for unraveling the tissue-specific functions and adaptive capabilities of these AMPs. The genome-wide identification of AMP genes in golden pompano holds significant importance in elucidating the immune defense mechanisms of this species, exploring novel antimicrobial peptide therapeutic strategies and promoting sustainable development in the aquaculture industry.

## Figures and Tables

**Table 1:** Comparison of the antimicrobial peptide (AMP) genes among *Trachinotus ovatus* and other fish.

AMP	*Trachinotus ovatus*	*Paralichthys olivaceus*	*Morone saxatilis*	*Scophthalmus maximus*	*Seriola dumerili*	*Seriola lalandi dorsalis*	*Acanthopagrus latus*	*Larimichthys crocea*	*Siniperca chuatsi*	*Channa argus*	*Oryzias latipes*	*Ctenopharyngodon idella*	*Oreochromis niloticus*	*Danio rerio*
Thrombin	79	70	80	72	82	96	93	93	75	62	76	104	126	108
Scolopendin	33	33	27	31	32	37	33	33	32	30	30	31	35	33
RegIIIgamma	30	23	32	32	38	37	39	49	40	35	43	41	82	58
Chemokine	22	20	23	20	26	26	31	32	34	29	28	38	70	73
BPTI	21	22	21	23	21	23	25	24	25	21	27	29	25	29
Ubiquitin	20	17	18	20	22	27	24	26	23	12	19	23	25	29
Histone H2A	16	12	12	28	26	31	27	27	18	17	20	55	25	43
Histone H3	15	7	4	16	14	19	18	20	10	13	16	42	22	22
RegIIIalpha	13	12	12	18	21	22	24	37	24	37	34	29	105	18
Hemoglobin	8	11	6	10	14	12	16	13	14	17	12	17	31	21
Histone H4	8	2	2	7	5	13	15	8	7	14	14	25	16	21
Histone H2B	8	3	3	18	14	19	20	22	10	16	14	55	17	36
Neuropeptide	8	10	9	9	11	8	10	13	9	11	9	8	10	11
β2-Microglobin	6	8	8	7	11	9	13	9	9	15	11	53	55	34
Antiproteinase	5	6	5	5	4	6	6	5	8	7	4	7	7	16
Hepcidin	5	3	3	3	6	5	13	7	4	2	6	2	14	2
Lysozyme	5	5	4	5	4	6	4	6	6	11	6	4	5	9
PLA2	5	5	5	7	4	5	5	6	5	4	3	4	4	4
Synuclein	4	2	3	4	4	4	4	4	4	3	4	4	4	4
GAPDH	3	2	3	3	3	3	3	3	3	3	2	2	3	2
Saposin	3	2	2	2	3	3	3	2	3	4	3	2	2	3
Amyloid	2	2	1	2	3	3	2	1	2	1	0	1	0	3
Ap-s	2	5	2	3	2	3	2	3	3	3	2	0	1	0
Defensin	2	2	3	3	3	3	3	2	3	5	4	3	7	6
LEAP-2	2	2	2	2	2	2	2	2	2	2	2	2	2	3
NK-lysin	2	1	4	2	2	1	1	4	0	0	1	2	4	10
Peptide_3910	2	2	2	2	2	2	2	3	2	2	2	1	2	1
S100A	2	0	2	1	2	2	0	2	0	2	2	0	3	0
Amylin-BP	1	1	1	1	1	1	1	1	1	1	1	1	1	1
CcAMP	1	1	1	1	1	1	1	1	1	1	1	1	1	1
cOT2	1	1	1	1	1	4	2	2	2	1	1	0	0	0
Ixodidin	1	1	1	2	2	0	2	2	1	1	0	1	1	2
pCM19	1	1	1	1	1	1	1	1	1	0	0	1	1	1
Piscidin	1	0	0	0	0	1	0	0	2	0	0	0	2	0
RNase	1	0	2	1	1	1	2	1	1	9	1	4	1	4
sOT2	1	2	2	2	2	2	1	1	1	1	2	3	3	2
Thymosin	1	2	2	1	3	3	2	1	2	1	0	5	2	4
Waprin	1	2	2	1	2	1	2	2	0	0	3	1	2	2
SPINK9-v1	0	3	2	3	4	4	4	4	3	3	2	6	3	8
Skin-PYY	0	1	1	1	1	1	1	1	1	1	2	1	1	2
YR26	0	1	0	0	0	0	0	0	0	0	0	0	0	1
Interleukin 26	0	1	0	0	0	0	1	1	0	0	0	0	0	0
Thrombocidin-1	0	0	1	1	1	1	1	1	1	0	0	1	1	0
sb-Moronecidin	0	0	1	0	0	0	0	0	0	0	0	0	0	0
Scygonadin2	0	0	0	0	1	1	0	0	0	0	0	0	0	0
Mytichitin-CB	0	0	0	0	0	1	0	0	0	3	0	0	0	0
Sushi peptide 1	0	0	0	0	0	0	1	0	0	0	1	1	0	1
Misgurin	0	0	0	0	0	0	0	0	0	1	0	0	0	0
Sonorensin	0	0	0	0	0	0	0	0	1	0	4	0	0	0
Fc-SWD	0	0	0	0	0	0	0	0	0	0	0	1	0	0
Kaliocin-1	0	0	0	0	0	0	0	0	0	0	0	0	1	1
Total	341	306	316	371	402	450	460	475	393	401	412	611	722	629

Note: The last row of each column indicates the total number of AMP genes obtained after comparing each species’ genome with the ADP3 database and subsequent filtering using an internal script. The remaining rows represent the number of AMP genes from different categories present in each species.

**Table 2:** The information of positive selection genes in *Trachinotus ovatus*.

AMP ID	Gene Name	Gene Description	dN/dS	FDR	*p*-Value
TCP.13	*hpn*	Hepsin	1.00	2.00 × 10^9^	8.79 × 10^9^
TCP.23	*tmprss6*	Transmembrane protease serine 6	221.989	0.00	0.00
Histone H2A.7	*macroh2a2*	Core histone macro-H2A.2	736.32	4.69 × 10^3^	0.001354
cgUbiquitin.13	*ubtd1*	Ubiquitin domain containing 1	284.27	0.00	0.00
Ap-s.2	*flnc*	Filamin-C-like	165.81	0.00	0.00

## Data Availability

Partial datasets presented in this study can be found inNCBI. The names of the repository and accession number can be found below: BioProject, accession number PRJNA871979. Additional analytical data, associated with unpublished work from our research team, is available upon direct request to the authors and has not been publicly disclosed.

## Supplementary Materials

The following supporting information can be downloaded at: https://www.mdpi.com/article/10.3390/md21100505/s1, File S1: The public Antimicrobial Peptides Database (ADP3); File S2: The identification of 341 potential AMP genes in the golden pompano; File S3: The results of PPI analysis.
